# Critical role of DEK and its regulation in tumorigenesis and metastasis of hepatocellular carcinoma

**DOI:** 10.18632/oncotarget.8565

**Published:** 2016-04-04

**Authors:** Le Yu, Xiaobin Huang, Wenfa Zhang, Huakan Zhao, Gang Wu, Fenglin Lv, Lei Shi, Yong Teng

**Affiliations:** ^1^ School of Life Sciences, Chongqing University, Chongqing 400044, PR China; ^2^ Third Affiliated Hospital, Third Military Medical University, Chongqing 400044, PR China

**Keywords:** DEK, HCC, isoform, migration, metastasis

## Abstract

Hepatocellular carcinoma (HCC) is one of the leading causes of cancer-related mortality globally. Therefore, it is quite essential to identify novel HCC-related molecules for the discovery of new prognostic markers and therapeutic targets. As an oncogene, DEK plays an important role in cell processes and participates in a variety of cellular metabolic functions, and its altered expression is associated with several human malignancies. However, the functional significance of DEK and the involved complex biological events in HCC development and progression are poorly understood. Here, combing the results from clinical specimens and cultured cell lines, we uncover a critical oncogenic role of DEK, which is highly expressed in HCC cells. DEK protein encompasses two isoforms (isoforms 1 and 2) and isoform 1 is the most frequently expressed DEK isoform in HCC cells. DEK depletion by using shRNA inhibited the cell proliferation and migration *in vitro* and suppressed tumorigenesis and metastasis in mouse models. Consistently, DEK overexpression regardless of which isoform produced the opposite effects. Further studies showed that DEK induced cell proliferation through upregulating cell cycle related CDK signaling, and promoted cell migration and EMT, at least in part, through the repression of β-catenin/E-cadherin axis. Interestingly, isoform 1 induced cell proliferation more efficiently than isoform 2, however, no functional differences existed between these two isoforms in cell migration. Together, our study indicates that DEK expression is required for tumorigenesis and metastasis of HCC, providing molecular insights for DEK-related pathogenesis and a basis for developing new strategies against HCC.

## INTRODUCTION

Hepatocellular carcinoma (HCC) is one of the most prevalent malignancies and the third most leading cause of cancer associated mortality in the world [1]. Given hepatocarcinogenesis is a complex and multi-step process which associates with various genetic changes [2, 3], it is of great significance to reveal the complicated molecular and cellular mechanisms of HCC development and progression in order to identify potential therapeutic targets for improving overall survival of HCC patients.

The DEK gene was originally discovered as a fusion partner in the (6;9)(p23;q34) chromosomal translocation in a subset of acute myelogenous leukemias [4]. The DEK gene encodes a nuclear protein that binds chromatin and is involved in various fundamental nuclear processes, including DNA damage repair [5], DNA replication [6], mRNA splicing [7], transcriptional regulation [8], differentiation [9], cell viability and motility [10]. The function of DEK has also been implicated in apoptosis, although with differing roles depending on the cellular context [11–13]. Upregulation of DEK may occur through copy gains [14] and its transcriptional activation is regulated by upstream regulators such as ERα [15], E2F [16], and NF-Y [17]. Emerging evidence suggests DEK has a dual role in repressing and activating transcription of target genes as a cofactor for transcriptional regulation [18–21].

High expression levels of DEK have been correlated with numerous human malignancies [22]. In high-grade neuroendocrine carcinoma of lung, overexpression of DEK is associated with tumor initiation activity and poor prognosis [23, 24]. In melanoma, inhibition of DEK is sufficient to drive melanoma cells into senescence whereas overexpression prolongs cellular lifespan [25, 26]. Moreover, DEK expression in gastric cancer correlates to tumor size, differentiation, clinical stage, disease-free survival, and overall survival rates [27]. Recent study has shown DEK promotes cellular proliferation through paracrine Wnt signaling in Ron receptor-positive breast cancer [28]. DEK also plays a significant role in hepatocyte differentiation and may serve as a useful prognostic marker for the staging of HCC [29–31]. However, its influence on HCC remains largely unknown.

Here, we report that elevated expression of DEK is critical for HCC progression. The loss of DEK inhibited cell proliferation and migration *in vitro*, delayed tumor growth in xenograft mouse model and suppressed mouse splenic vein metastasis. We identified that isoform 1 was predominantly expressed in HCC cells and the absence of amino acids 49-82 in DEK protein was required for cell proliferation, but not for cell motility. We also present new evidence that DEK facilitated HCC cell proliferation through modulating cell cycle related genes. In addition, DEK promoted HCC cell migration and EMT probably, at least in part, through the regulation of β-catenin/E-cadherin signaling. Our study provides new insights into the downstream molecular events of DEK signaling and unveils the function of DEK in tumorigenesis and metastasis of HCC.

## RESULTS

### DEK is highly expressed in HCC cells and tissue samples

**Figure 1:**
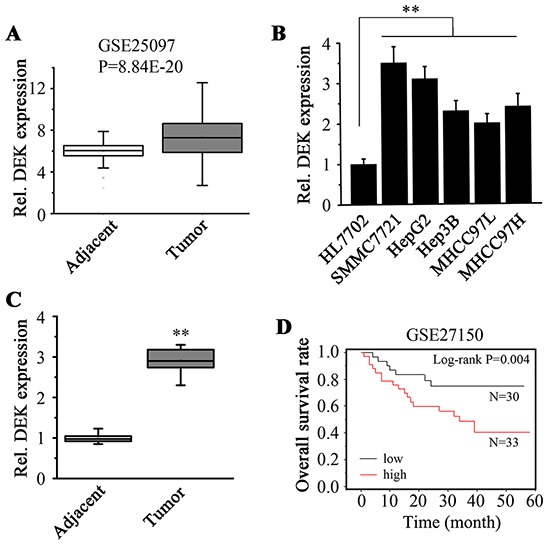
DEK expression is elevated in HCC tissues and cell lines **A.** Gene expression data obtained from GSE25097 dataset were used to analyze DEK expression in tumor and adjacent samples. Units for Y-axis are absolute expression value from microarray data. **B.** RT-qPCR was used to determine the DEK expression in HCC cells and liver normal cells. **C.** Tumor and adjacent non-tumor tissue pairs from patients with HCC were collected and RT-qPCR was performed to measure the DEK expression. **D.** Kaplan-Meier analysis was performed to determine the correlation between the DEK expression and overall survival of HCC patients. Units for Y-axis are survival percentages of HCC patients. Data are represented as mean ± SEM, Results of RT-qPCR presented represent the mean of triplicate experiments ± SEM. *P<0.05; **P<0.01.

To explore the role of DEK in human HCC, we assessed the DEK gene expression in HCC clinical samples using microarray data from Gene Expression Omnibus (GEO), which showed that the expression levels of DEK in tumor tissues were elevated compared with the adjacent non-tumor tissues (Figure 1A). We then determined DEK expression in HCC cell lines and clinical specimens using RT-qPCR assays. mRNA levels of DEK in HCC cell lines (SMMC7721, HepG2, Hep3B, MHCC97L and MHCC97H) were noticeably higher than those in immortalized liver cells HL7702 (Figure 1B). Similarly, higher expression levels of DEK were observed in 19 pairs of HCC tissue samples compared with those in corresponding adjacent non-tumor tissue samples (Figure 1C). We analyzed survival with gene expression data using the annotated GEO dataset. Kaplan-Meier estimate showed high DEK expression levels were strongly associated with the low patient survival in the carcinomas (Figure 1D), which supports a notion that DEK plays a critical role in HCC progression.

### DEK depletion inhibits cell proliferation and migration in HCC cells *in vitro*

**Figure 2:**
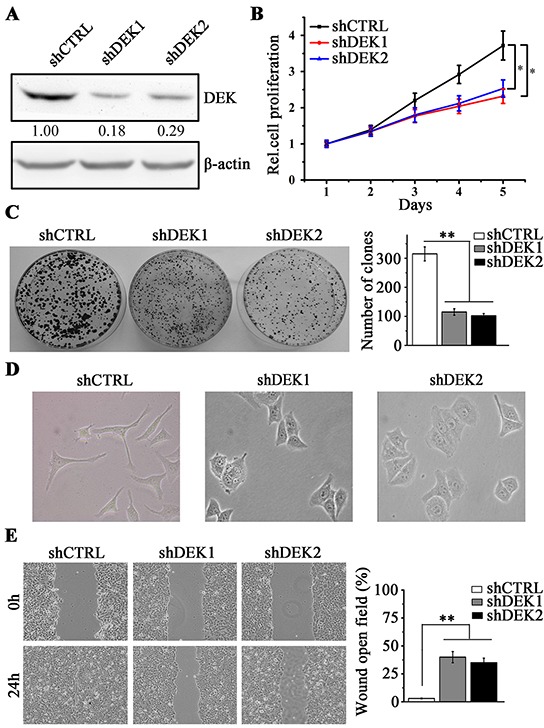
DEK knockdown inhibits cell proliferation and migration in SMMC7721 cells **A.** Western blotting was performed to detect the knockdown effect by shRNA against DEK (shDEK-1 and shDEK-2). The non-target shRNA-expressing cells (shCTRL) were the knockdown control cells. **B.** MTS assay was performed to determine the cell proliferation when DEK was knocked down in SMMC7721 cells. **C.** Clonogenic assay was performed to measure the capacity of colony formation when DEK was depleted. 1×10^3^ SMMC7721 cells were seeded in 6-well-plate to form colonies in 3 weeks and colonies with no less than 50 cells/colony were counted. Quantitation of colony number was shown in the right panel. **D.** Morphology showing knockdown of DEK in SMMC7721 cells reverts typically mesenchymal morphology to epithelial characteristics. **E.** Wound-healing assay was employed to determine the migration of SMMC7721 cells in response to DEK depletion. Cells were monitored within 24 hours to evaluate the rate of migration into the scratched area. Results presented represent the mean of triplicate experiments ± SEM. *P<0.05; **P<0.01.

To investigate the impact of DEK in HCC, we knocked down DEK in high-invasive HCC cell line SMMC7721. The cells were transduced with non-targeting shRNA (shCTRL) or two different DEK-specific lentiviral shRNA (shDEK1 and shDEK2), and the knockdown effect was confirmed by western blotting (Figure 2A). We found that DEK knockdown led to a remarkable inhibition in cell proliferation as determined by MTS (Figure 2B). Depletion of DEK in these cells also resulted in a significant decrease in colony-forming capacity compared with those in the knockdown control cells (Figure 2C). SMMC7721 cells are typically mesenchymal-like. Interestingly, when DEK was knocked down, these cells rendered an epithelial morphology (Figure 2D) and lost migratory capability (Figure 2E). These results suggest that DEK depletion suppresses cell migration may through diminishing EMT traits in HCC cells.

### DEK depletion suppresses tumorigenesis and metastasis in HCC cells *in vivo*

**Figure 3:**
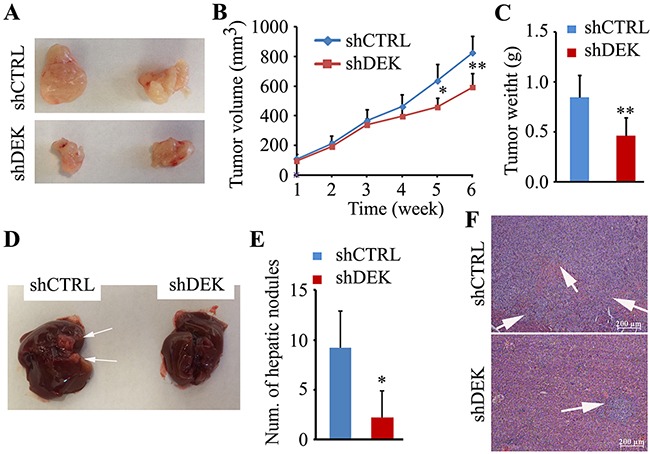
Knockdown of DEK suppresses tumor growth and metastasis *in vivo* **A.** Representative images showing xenograft tumors at day 42 post subcutaneous injection (n=5). **B, C.** Tumor sizes measured and depicted as tumor volume (B) or tumor weight (C). **D.** Representative images showing metastatic liver nodules in nude mice by splenic-vein injection of DEK knockdown SMMC7721 cells and the knockdown control cells. The arrows indicate the metastatic tumor on the surface of the liver. **E.** The numbers of nodules were quantified on nude mice livers (n=5). **F.** H&E staining was performed on serial sections of metastatic tumors and normal liver (×200). The arrows show the tumor foci in mouse liver section. Data are represented as mean ± SEM. **P<0.01.

Having mechanistically deciphered the oncogenic properties of DEK *in vitro*, we next examined whether silencing DEK affects tumorigenesis and metastasis *in vivo*. In mouse xenograft model, DEK-depleted xenografts were significantly smaller in volume and weight (Figure 3A–3C). The nude mice splenic vein metastasis models showed that mice injected with the DEK knockdown SMMC7721 cells had less metastatic nodules on the liver surface than mice injected with the knockdown control cells (Figure 3D and 3E). Histological analysis further showed that reduced DEK expression in SMMC7221 resulted in fewer and smaller tumor foci in liver section compared to the knockdown control (Figure 3F).

### DEK promotes cell proliferation and migration in HCC cells

DEK gene encodes two distinct proteins, produced by alternative splicing of the DEK gene transcript [32]. Isoform 1 is a full-length DEK form, while isoform 2 is a truncated short form (missing amino acids 49-82, Supplementary Figure S1A) results from an alternative splicing of encoding mRNA, which gives rise to a deletion in its fragment shown to be necessary for the positive DNA supercoiling activity of DEK. The role of these two isoforms in HCC progression is unclear, therefore, we sought to determine their presence in HCC cells and tissue samples using isoform-specific primers. DEK isoform 1 was found in all HCC cell lines examined in our study as well as HeLa cells and breast cancer MCF7 and MDA-MB-231 cells (Supplementary Figure S1B and S1C), whereas isoform 2 cannot be detected in any of tested cell lines. We then examined DEK isoforms 1 and 2 in HCC clinical samples. DEK isoform 2 was only observed in 1 out of 19 patients with HCC (5.26%) (Supplementary Figure S1D), suggesting isoform 1 is predominantly expressed in HCC.

**Figure 4:**
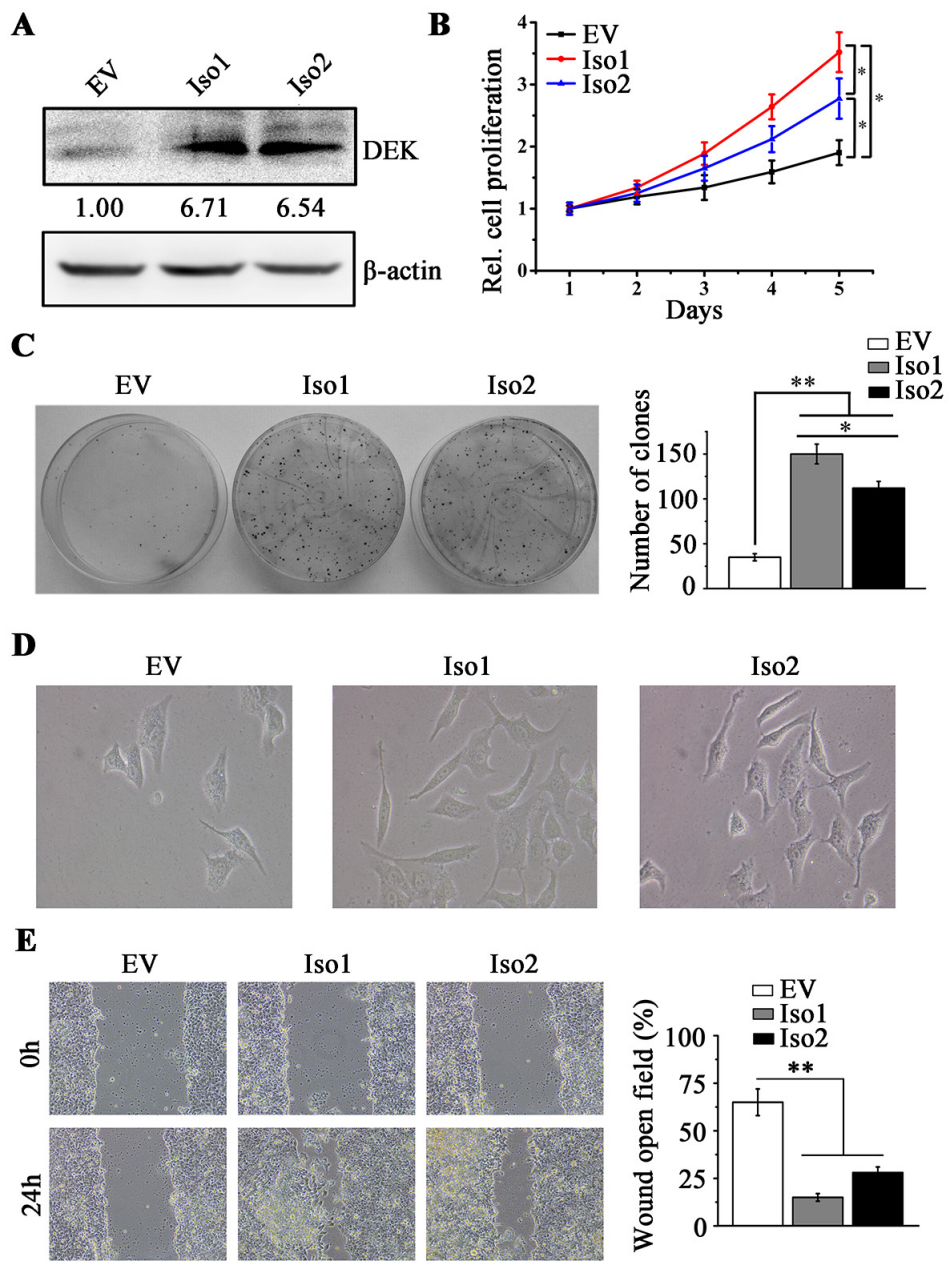
DEK overexpression promotes cell proliferation and migration in MHCC97L cells **A.** Western blotting was performed to determine the effect of DEK overexpression. **B.** MTS assay was performed to determine the cell proliferation when DEK isoform 1 (Iso1) or isoform 2 (Iso2) was ectopically expressed in MHCC97L cells. Cells overexpressing empty vector (EV) were the control cells. **C.** Clonogenic assay was performed to measure the capacity of colony formation when DEK was overexpressed. 1×10^3^ MHCC97L cells were seeded in 6-well-plate to form colonies in 2 weeks and colonies with no less than 50 cells/colony were counted. Quantitation of colony number was shown in the right panel. **D.** Morphology showing DEK overexpression facilitates EMT in MHCC97L cells **E.** Wound-healing assay was employed to determine the migration of MHCC97L cells in response to DEK overexpression. Cells were monitored within 24 hours to evaluate the rate of migration into the scratched area. Results presented represent the mean of triplicate experiments ± SEM. *P<0.05; **P<0.01.

We individually introduced exogenous isoforms 1 and 2 into low-invasive MHCC97L cells and the effect of DEK overexpression was confirmed by RT-qPCR (Supplementary Figure S2) and western blotting (Figure 4A). Interestingly, increased cell proliferation and colony-forming units was observed regardless of which isoform overexpressed (Figure 4B and 4C). However, isoform 1 overexpression promoted cell proliferation and colony formation more efficiently than overexpression of isoform 2 (Figure 4B and 4C). We next determined the cell morphological change and migration when DEK was overexpressed. Forced expression of either isoforms 1 or 2 in MHCC97L cells facilitated epithelial cells to revert to a mesenchymal phenotype and enhanced the migration potential (Figure 4D and 4E). There was no difference between these two isoforms in cell migration and EMT (Figure 4D and 4E). These findings indicate that the absence of amino acids 49-82 in DEK protein limits its function in cell proliferation but not cell motility.

### DEK promotes cell proliferation through the regulation of cell cycle related genes

**Figure 5:**
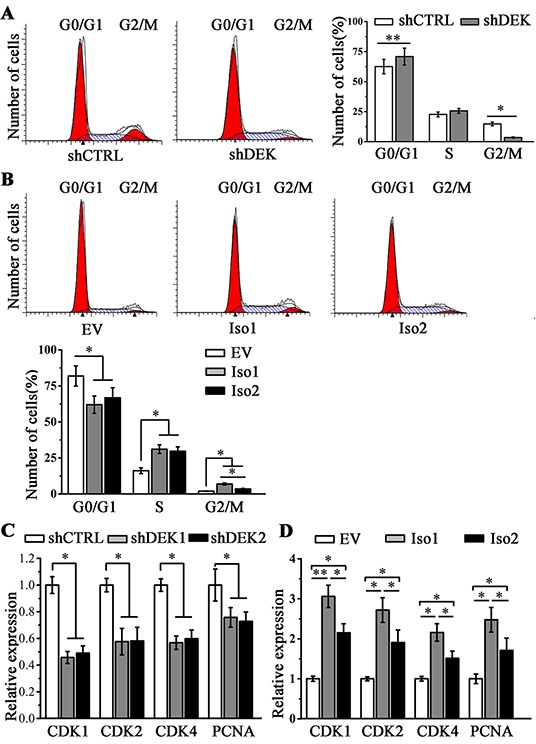
DEK promotes cell proliferation through the regulation of cell cycle related genes **A, B.** Flow cytometry analysis was performed to determine the cell cycle progression when DEK was depleted (A) or overexpressed (B). **C, D.** RT-qPCR was performed to determine the expression of CDK1, CDK2, CDK4 and PCNA genes when DEK was depleted (C) or overexpressed (D). Results presented represent the mean of triplicate experiments ± SEM. *P<0.05; **P<0.01.

To explore a potential mechanism underlying the role of DEK in cancer cell proliferation, we assessed the proportion of cells in each phase of the cell cycle when DEK expression was depleted or overexpressed. Flow cytometry analysis showed that DEK depletion inhibited SMMC7721 cell growth by causing progressive cell cycle arrest (Figure 5A). In contrast, ectopically expressing DEK isoform 1 or 2 induced cell cycle progression in MHCC97L cells (Figure 5B). These observation promoted us to analyze gene expression correlation using HCC database from the Cancer Genome Atlas (TCGA). Pearson correlation coefficient analysis by cBioPortal showed that the expression levels between DEK and several cell cycle related genes were in a strongly positive linear association (Supplementary Figure S3). To validate above bio-statistical analysis, CDK1, CDK2, CDK4 and PCNA were selected for RT-qPCR assays. DEK knockdown in SMMC7721 cells led to a remarkable reduction in their expression (Figure 5C), and an opposite effect was observed when DEK was overexpressed in MHCC97L cells (Figure 5D). However, isoform 1 enhanced the expression levels of these genes and facilitated the entrance into mitosis (the M phase) more significantly than isoform 2 (Figure 5B and 5D).

### DEK promotes cell migration through the down-regulation of E-cadherin

**Figure 6:**
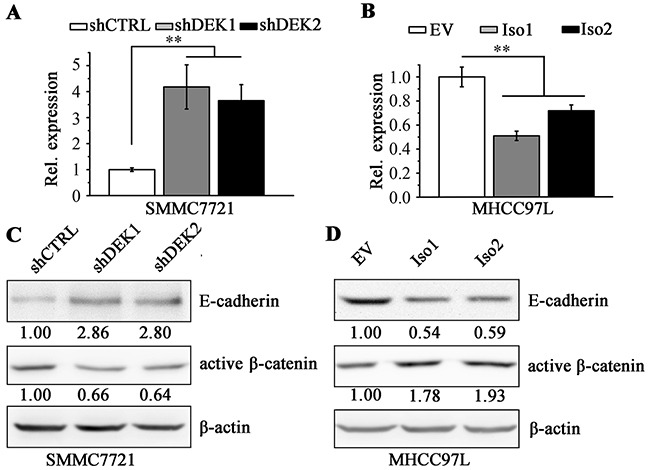
DEK negatively regulates E-cadherin through enhancing active β-catenin **A, B.** RT-qPCR was performed to determine the E-cadherin expression at mRNA levels when DEK was depleted (A) or overexpressed (B) in HCC cells. **C, D.** Western blotting was performed to measure the protein levels of E-cadherin and enhances active β-catenin when DEK was depleted (C) or overexpressed (D) in HCC cells. Data are represented as mean ± SEM. *P<0.05; **P<0.01.

We next asked if DEK regulates the expression of genes involved in cell motility. Pearson correlation coefficient analysis by cBioPortal showed that the expression levels of DEK and E-cadherin/CDH1 were in a negative linear association (Supplementary Figure S4). E-cadherin is an epithelial cell adhesion molecule and loss of its expression prominently associates with tumor invasiveness, metastatic dissemination and poor patient prognosis [33–35]. Knockdown of DEK in SMMC7721 cells led to dramatically increased E-cadherin expression compared with the knockdown control cells (Figure 6A and 6C), whereas, overexpression of DEK decreased E-cadherin levels in MHCC97L cells (Figure 6B and 6D). We did not identify that the suppression effect of isoform1 on E-cadherin expression differed from isoform 2, which may explain the results of cell migration (Figure 4E). The amount of active β-catenin leads to a transcriptional repression of E-cadherin [36, 37]. We then determine the active β-catenin in DEK knockdown SMMC7721 cells, which showed lower levels than that in the knockdown control cells (Figure 6C). Conversely, elevated active β-Catenin were detected in DEK-overexpressing MHCC97L cells (Figure 6D), demonstrating DEK promotes EMT and migration of HCC cells through activating β-catenin to repress E-cadherin expression.

## DISCUSSION

DEK has been implicated in two possibly related functions: alterations in the topology of DNA and chromatin for affecting DNA replication and RNA processing [6, 38]. Recent studies indicate that DEK plays crucial roles in numerous cancers [22–31], while its function in HCC is rarely elucidated. We found that knockdown of DEK in MHCC97H and HepG2 cells also inhibited cell proliferation and migration (Supplementary Figure S5), although the suppression effects were more efficient in SMCC7721 when DEK was depleted. Our *in vitro* and *in vivo* studies were performed with the established HCC cell lines, but the observations together with the previous clinical report [29–31] that high DEK abundance in HCC was strongly correlated with poor overall survival of HCC patients, suggesting a general oncogenic role of DEK in HCC development and progression.

Cyclin/cyclin-dependent kinases (CDKs) bind to cyclin to form active CDK-cyclin protein complexes (CDKC), controlling cell division cycle and proliferation [39]. DEK expression has been shown to correlate with cyclinD1 expression [28]. Here, we showed that knockdown of DEK in high-invasive HCC cells inhibited cell proliferation through promoting cell cycle arrest. These findings are not surprised because the expression levels of CDK1, 2 and 4, the three members of CDKs directly involved in cell cycle regulation [40, 41], dramatically decreased when DEK was knocked down. PCNA is one of DEK coexpressed genes in HCC. PCNA protein presents in quaternary complexes with CDKs and involves in cell cycle regulatory network [42]. In this study, PCNA was also downregulated in DEK depletion cells. Therefore, DEK may facilitate HCC cell proliferation through upregulation of CDKs signaling.

We also report the novel association of DEK with EMT through suppressing the E-cadherin expression. Evidence including changes in cell morphology, reversal of EMT characteristics, loss of clonogenecity and migration potential upon DEK knockdown suggests the positive role of DEK in tumorigenesis and metastasis of HCC. It is well-established that loss of E-cadherin expression is a hallmark of EMT and its transcription is negatively regulated by active β-catenin [43, 44]. DEK has been shown to promote breast cancer cell invasion by inducing β-catenin activity [10]. The data shown here further demonstrate that DEK-dependent migration and EMT occurs at least partly via β-catenin/E-cadherin signaling in HCC cells.

DEK protein encompasses two isoforms (isoforms 1 and 2). In humans, isoforms 1 and 2 represent splice variants of DEK that are encoded by a DEK gene [32]. A number of investigations have mostly focused on understanding the role of isoform 1 in cancer development. The first report revealing DEK isoform 2 was in urine of humans who suffered from bladder cancer disorder [45]. The underlying mechanistic basis for this finding is unclear but it is unlikely that only isoform 2 can be secreted or released during the pathogenesis of bladder cancer. Our study represents the first time for the existence of isoform 2 in HCC cells, although isoform 1 is predominantly expressed. Interestingly, isoform 2 can also induce HCC cell proliferation, but its effect is less potent when compared with isoform 1. No functional differences exist between these two isoforms in cell migration. These phenotypes may rely on the function of the deletion region (49-82aa) in isoform 2. Since immunosuppressive agents, such as dexamethasone and cyclosporine A, can block the secretion of DEK by monocyte-derived macrophages [46], our results suggest that targeting DEK may be a potential approach to combat HCC.

## MATERIALS AND METHODS

### Human HCC specimens and cell lines

Nineteen primary HCC tissues with paired adjacent normal liver tissues were from Third Affiliated Hospital of Third Military Medical University (Chongqing, China). Informed consent was given by all of the patients and all samples were histologically confirmed before analysis. HL7702, HepG2, MHCC97L, MHCC97H, Hep3B, SMMC7721, Hela, MCF7 and MDA-MB-231 cells were directly obtained from the Cell Bank of Type Culture Collection of Chinese Academy of Sciences (Shanghai, China) and passage <5 were used in this study. All the cell lines are maintained according to the supplier's instructions.

### RT-PCR, RT-qPCR and western blotting analysis

Total RNA was extracted from cells and tissues using Trizol (Life technologies, NY) according to the manufacturer's instructions. Upon isolating RNA, DNase I was treated to eliminate contaminating genomic DNA. RevertAid First Strand cDNA Synthesis Kit (Life technologies) was used for cDNA synthesis.

The mRNA expression levels of DEK isoform in the tumor cells and tissues were detected with RT-PCR. The primer sequences were: DEK-forward 5′-AGG AGG AGG AGG AGG AAA-3′, DEK-reverse 5′- CAC TGA ACT GAC CCA CAT T-3′; GAPDH-forward 5′-GTG AAG GTC GGT GTC AAC GGA TTT-3′ and GAPDH-reverse 5′-CAC AGT CTT CTG AGT GGC AGT GAT-3′. Quantification of mRNA using qPCR performed using GoTaq qPCR Master Mix (Promega) on a BioRad CFX96 (Bio-Rad, CA). Gene expression levels were analyzed using the delta Ct method and normalized against β-actin. The gene-specific primers used in RT-qPCR were listed in Table 1. Western blotting assays were performed according to a standard protocol and densitometric volume of the target bands was quantified using Fiji software.

**Table 1:** Primers for RT-qPCR in this study

Gene name	Forward (5′-3′)	Reverse (5′-3′)
DEK	GGAGATCCGCCCAGATGGCTAC	GGCCTCCAGTCCGGTGACAAGC
E-cadherin	TGAAGGTGACAGAGCCTCTGGAT	TGAAGGTGACAGAGCCTCTGGAT
CDK1	GGATGTGCTTATGCAGGATTCC	CATGTACTGACCAGGAGGGATAG
CDK2	CCAGGAGTTACTTCTATGCCTGA	TTCATCCAGGGGAGGTACAAC
CDK4	TCAGCACAGTTCGTGAGGTG	GTCCATCAGCCGGACAACAT
PCNA	CCTGCTGGGATATTAGCTCCA	CAGCGGTAGGTGTCGAAGC
β-actin	ATAGCACAGCCTGGATAGCAACGTAC	CACCTTCTACAATGAGCTGCGTGTG

### Antibodies and constructs

DEK and β-Actin antibodies were purchased from Abcam (Cambridge, MA) and Sigma (St Louis, MO), respectively. Antibodies against E-cadherin and non-phospho β-Catenin (active) were from Cell Signaling (Beverly, MA). The following primers were used to amplify the full-length of DEK isoform 1: forward 5′-GGG GTA CCC CAT GTC CGC CTC GGC CCC TGC TGC-3′; reverse 5′-GCT CTA GAG CTC AAG AAA TTA GCT CTT TTA CAG-3′. Overlap extension PCR was used to amplify DEK isoform 2 . Briefly, the segment 1 (1-144nt) and segment 2 (145-1026nt) were amplified respectively using the following primers: segment 1-forward 5′-GGG GTA CCC CAT GTC CGC CTC GGC CCC TGC TGC-3′; segment 1-reverse 5′-GCC CCT TTC CTT TTT CCT CCT CCT CCT CCT CCT-3′; segment 2-forward 5′-GCT CTA GAG CTC AAG AAA TTA GCT CTT TTA CAG-3′; segment 2-reverse 5′-GGA GGA AAA AGG AAA GGG GCA GAA ACT TTG TGA-3′. The full-length of isoform 2 was then amplified from mixed templates of segment 1 and 2 using the same primers for isoform 1. The isoforms 1 and 2 were cloned into pcDNA3.1 (+) expression vector (Invitrogen, USA) using *KpnI* and *XbaI* sites. To knock down DEK, lentiviral vectors harboring shRNAs targeting DEK (shDEK1 and shDEK2) were obtained from GeneCopoeia (Rockville, MD) and packaging of the DEK constructs in pseudoviral particles was performed in 293T cells using Lenti-Pac™ FIV Expression Packaging Kit (GeneCopoeia). Non-target shRNA (shCTRL) was used as a negative control in this study.

### Cell proliferation and clonogenic assays

For cell proliferation, 1×10^4^ cells were seeded in 96-well-plate supplied in triplicates and the CellTiter 96® AQ_ueous_ One Solution Cell Proliferation Assay kit (Promega, WI) was used to monitor the cell proliferation rate continuously until 96 hours. For clonogenic assay, 1×10^3^ cells were seeded in 6-well-plate to form colonies in 2-3 weeks. Colonies were fixed with glutaraldehyde (6.0% v/v), stained with crystal violet (0.5% w/v) and counted using a stereomicroscope. Colonies with no less than 50 cells/colony were counted.

### Cell cycle analysis

DNA content in cells was quantitated by flow cytometry of cells stained with propidium iodide (PI). Briefly, cancer cells were collected and washed twice with ice-cold PBS followed by fixed with 70% alcohol overnight. The cells were stained with 1mg/mL PI (Promega) in the presence of 1% RNase A (Promega) for 30 min and cell populations in the G0/G1, S, and G2/M phases were measured by a flow cytometry (Becton Dickinson, CA). Data were analyzed with the Mod-FitLT software (Becton Dickinson).

### Wound-healing assay

For this assay, confluent monolayers were starved overnight and a single scratch wound was created by dragging a 10 μl plastic pipette tip across the cell surface. The area of a defined region within the scratch was measured using ImageJ software. The extent to which the wound had closed over 24 hours was calculated and expressed as a percentage of the difference between times 0 and 24 h.

### *In vivo* tumorigenesis and splenic vein metastasis assay

Six-week-old nude mice were inoculated subcutaneously in the right hind flank with 5×10^6^ cells per 100 μl suspended in dilute Matrigel 1:1 in ice cold PBS. Tumor development was monitored over a period of 6 weeks before the mice were euthanized for further analysis. Tumor volume (mm^3^) is measured with calipers and calculated as (W^2^ ×L)/2, where W is width and L is length. For splenic vein metastasis assay, cells were injected into the splenic vein of 8-week-old nude mice at 2×10^6^ cells per injection site. The mice were sacrificed after 6 weeks and the numbers of surface liver metastases were counted. Liver was then removed and harvested for histological analysis by hematoxylin and eosin (H&E) staining. All procedures involving animals were performed in accordance with the institutional animal welfare guidelines of Chongqing University.

### Statistical analysis

Statistical analyses were performed using Student's t test and one-way ANOVA by SPSS18.0 for Windows. Data were presented as mean ± SEM of at least three independent experiments. DEK gene expression data were obtained from GEO database. Pearson's correlation coefficient was used to determine the correlation between DEK and other interested genes calculated from TCGA data by cBioportal (www.cbioportal.org) [47, 48]. A P-value of 0.05 or less was considered to be significant.

## SUPPLEMENTARY FIGURES



## References

[1] Forner A, Llovet JM, Bruix J (2012). Hepatocellular carcinoma. Lancet.

[2] Zhang D, Lim SG, Koay ES (2007). Proteomic identification of down-regulation of oncoprotein DJ-1 and proteasome activator subunit 1 in hepatitis B virus-infected well-differentiated hepatocellular carcinoma. International journal of oncology.

[3] Thorgeirsson SS, Lee JS, Grisham JW (2006). Molecular prognostication of liver cancer: end of the beginning. Journal of hepatology.

[4] von Lindern M, van Baal S, Wiegant J, Raap A, Hagemeijer A, Grosveld G (1992). Can, a putative oncogene associated with myeloid leukemogenesis, may be activated by fusion of its 3′ half to different genes: characterization of the set gene. Molecular and cellular biology.

[5] Kavanaugh GM, Wise-Draper TM, Morreale RJ, Morrison MA, Gole B, Schwemberger S, Tichy ED, Lu L, Babcock GF, Wells JM, Drissi R, Bissler JJ, Stambrook PJ, Andreassen PR, Wiesmuller L, Wells SI (2011). The human DEK oncogene regulates DNA damage response signaling and repair. Nucleic Acids Res.

[6] Alexiadis V, Waldmann T, Andersen J, Mann M, Knippers R, Gruss C (2000). The protein encoded by the proto-oncogene DEK changes the topology of chromatin and reduces the efficiency of DNA replication in a chromatin-specific manner. Genes Dev.

[7] McGarvey T, Rosonina E, McCracken S, Li Q, Arnaout R, Mientjes E, Nickerson JA, Awrey D, Greenblatt J, Grosveld G, Blencowe BJ (2000). The acute myeloid leukemia-associated protein, DEK, forms a splicing-dependent interaction with exon-product complexes. J Cell Biol.

[8] Sammons M, Wan SS, Vogel NL, Mientjes EJ, Grosveld G, Ashburner BP (2006). Negative regulation of the RelA/p65 transactivation function by the product of the DEK proto-oncogene. J Biol Chem.

[9] Wise-Draper TM, Morreale RJ, Morris TA, Mintz-Cole RA, Hoskins EE, Balsitis SJ, Husseinzadeh N, Witte DP, Wikenheiser-Brokamp KA, Lambert PF, Wells SI (2009). DEK proto-oncogene expression interferes with the normal epithelial differentiation program. Am J Pathol.

[10] Privette Vinnedge LM, McClaine R, Wagh PK, Wikenheiser-Brokamp KA, Waltz SE, Wells SI (2011). The human DEK oncogene stimulates beta-catenin signaling, invasion and mammosphere formation in breast cancer. Oncogene.

[11] Hua Y, Hu H, Peng X (2009). Progress in studies on the DEK protein and its involvement in cellular apoptosis. Sci China C Life Sci.

[12] Kim DW, Chae JI, Kim JY, Pak JH, Koo DB, Bahk YY, Seo SB (2009). Proteomic analysis of apoptosis related proteins regulated by proto-oncogene protein DEK. J Cell Biochem.

[13] Lee KS, Kim DW, Kim JY, Choo JK, Yu K, Seo SB (2008). Caspase-dependent apoptosis induction by targeted expression of DEK in Drosophila involves histone acetylation inhibition. J Cell Biochem.

[14] Grasemann C, Gratias S, Stephan H, Schuler A, Schramm A, Klein-Hitpass L, Rieder H, Schneider S, Kappes F, Eggert A, Lohmann DR (2005). Gains and overexpression identify DEK and E2F3 as targets of chromosome 6p gains in retinoblastoma. Oncogene.

[15] Privette Vinnedge LM, Ho SM, Wikenheiser-Brokamp KA, Wells SI (2012). The DEK oncogene is a target of steroid hormone receptor signaling in breast cancer. Plos One.

[16] Carro MS, Spiga FM, Quarto M, Di Ninni V, Volorio S, Alcalay M, Muller H (2006). DEK Expression is controlled by E2F and deregulated in diverse tumor types. Cell Cycle.

[17] Sitwala KV, Adams K, Markovitz DM (2002). YY1 and NF-Y binding sites regulate the transcriptional activity of the dek and dek-can promoter. Oncogene.

[18] Hollenbach AD, McPherson CJ, Mientjes EJ, Iyengar R, Grosveld G (2002). Daxx and histone deacetylase II associate with chromatin through an interaction with core histones and the chromatin-associated protein Dek. J Cell Sci.

[19] Ko SI, Lee IS, Kim JY, Kim SM, Kim DW, Lee KS, Woo KM, Baek JH, Choo JK, Seo SB (2006). Regulation of histone acetyltransferase activity of p300 and PCAF by proto-oncogene protein DEK. Febs Lett.

[20] Kim DW, Kim JY, Choi S, Rhee S, Hahn Y, Seo SB (2010). Transcriptional regulation of 1-cys peroxiredoxin by the proto-oncogene protein DEK. Mol Med Rep.

[21] Campillos M, Garcia MA, Valdivieso F, Vazquez J (2003). Transcriptional activation by AP-2alpha is modulated by the oncogene DEK. Nucleic Acids Res.

[22] Wise-Draper TM, Mintz-Cole RA, Morris TA, Simpson DS, Wikenheiser-Brokamp KA, Currier MA, Cripe TP, Grosveld GC, Wells SI (2009). Overexpression of the cellular DEK protein promotes epithelial transformation in vitro and in vivo. Cancer Res.

[23] Shibata T, Kokubu A, Miyamoto M, Hosoda F, Gotoh M, Tsuta K, Asamura H, Matsuno Y, Kondo T, Imoto I, Inazawa J, Hirohashi S (2010). DEK oncoprotein regulates transcriptional modifiers and sustains tumor initiation activity in high-grade neuroendocrine carcinoma of the lung. Oncogene.

[24] Wang X, Lin L, Ren X, Lin Z, Li Z, Li C, Jin T (2014). High expression of oncoprotein DEK predicts poor prognosis of small cell lung cancer. International journal of clinical and experimental pathology.

[25] Wise-Draper TM, Allen HV, Thobe MN, Jones EE, Habash KB, Munger K, Wells SI (2005). The human DEK proto-oncogene is a senescence inhibitor and an upregulated target of high-risk human papillomavirus E7. J Virol.

[26] Wise-Draper TM, Allen HV, Jones EE, Habash KB, Matsuo H, Wells SI (2006). Apoptosis inhibition by the human DEK oncoprotein involves interference with p53 functions. Mol Cell Biol.

[27] Piao J, Shang Y, Liu S, Piao Y, Cui X, Li Y, Lin Z (2014). High expression of DEK predicts poor prognosis of gastric adenocarcinoma. Diagn Pathol.

[28] Privette Vinnedge LM, Benight NM, Wagh PK, Pease NA, Nashu MA, Serrano-Lopez J, Adams AK, Cancelas JA, Waltz SE, Wells SI (2015). The DEK oncogene promotes cellular proliferation through paracrine Wnt signaling in Ron receptor-positive breast cancers. Oncogene.

[29] Lu ZL, Luo DZ, Wen JM (2005). Expression and significance of tumor-related genes in HCC. World journal of gastroenterology.

[30] Kondoh N, Wakatsuki T, Ryo A, Hada A, Aihara T, Horiuchi S, Goseki N, Matsubara O, Takenaka K, Shichita M, Tanaka K, Shuda M, Yamamoto M (1999). Identification and characterization of genes associated with human hepatocellular carcinogenesis. Cancer research.

[31] Yi HC, Liu YL, You P, Pan JS, Zhou JY, Liu ZJ, Zhang ZY (2015). Overexpression of DEK gene is correlated with poor prognosis in hepatocellular carcinoma. Molecular medicine reports.

[32] Privette Vinnedge LM, Kappes F, Nassar N, Wells SI (2013). Stacking the DEK: from chromatin topology to cancer stem cells. Cell Cycle.

[33] Fougner SL, Lekva T, Borota OC, Hald JK, Bollerslev J, Berg JP (2010). The expression of E-cadherin in somatotroph pituitary adenomas is related to tumor size, invasiveness, and somatostatin analog response. The Journal of clinical endocrinology and metabolism.

[34] Wells A, Yates C, Shepard CR (2008). E-cadherin as an indicator of mesenchymal to epithelial reverting transitions during the metastatic seeding of disseminated carcinomas. Clinical & experimental metastasis.

[35] Chu K, Boley KM, Moraes R, Barsky SH, Robertson FM (2013). The paradox of E-cadherin: role in response to hypoxia in the tumor microenvironment and regulation of energy metabolism. Oncotarget.

[36] Nelson WJ, Nusse R (2004). Convergence of Wnt, beta-catenin, and cadherin pathways. Science.

[37] MacDonald BT, Tamai K, He X (2009). Wnt/beta-catenin signaling: components, mechanisms, and diseases. Developmental cell.

[38] Riveiro-Falkenbach E, Soengas MS (2010). Control of tumorigenesis and chemoresistance by the DEK oncogene. Clin Cancer Res.

[39] Sarita Rajender P, Ramasree D, Bhargavi K, Vasavi M, Uma V (2010). Selective inhibition of proteins regulating CDK/cyclin complexes: strategy against cancer--a review. Journal of receptor and signal transduction research.

[40] Besson A, Dowdy SF, Roberts JM (2008). CDK inhibitors: cell cycle regulators and beyond. Developmental cell.

[41] Gerard C, Goldbeter A (2009). Temporal self-organization of the cyclin/Cdk network driving the mammalian cell cycle. Proceedings of the National Academy of Sciences of the United States of America.

[42] Li Y, Jenkins CW, Nichols MA, Xiong Y (1994). Cell cycle expression and p53 regulation of the cyclin-dependent kinase inhibitor p21. Oncogene.

[43] Li Y, Welm B, Podsypanina K, Huang S, Chamorro M, Zhang X, Rowlands T, Egeblad M, Cowin P, Werb Z, Tan LK, Rosen JM, Varmus HE (2003). Evidence that transgenes encoding components of the Wnt signaling pathway preferentially induce mammary cancers from progenitor cells. Proceedings of the National Academy of Sciences of the United States of America.

[44] Heuberger J, Birchmeier W (2010). Interplay of cadherin-mediated cell adhesion and canonical Wnt signaling. Cold Spring Harbor perspectives in biology.

[45] Datta A, Trama Jason (2014). Detection of a specific DEK isoform as a urine-based biomarker for bladder cancer.

[46] Pease NA, Wise-Draper T, Privette Vinnedge L (2015). Dissecting the Potential Interplay of DEK Functions in Inflammation and Cancer. J Oncol.

[47] Gao J, Aksoy BA, Dogrusoz U, Dresdner G, Gross B, Sumer SO, Sun Y, Jacobsen A, Sinha R, Larsson E, Cerami E, Sander C, Schultz N (2013). Integrative analysis of complex cancer genomics and clinical profiles using the cBioPortal. Science signaling.

[48] Cerami E, Gao J, Dogrusoz U, Gross BE, Sumer SO, Aksoy BA, Jacobsen A, Byrne CJ, Heuer ML, Larsson E, Antipin Y, Reva B, Goldberg AP, Sander C, Schultz N (2012). The cBio cancer genomics portal: an open platform for exploring multidimensional cancer genomics data. Cancer discovery.

