# Supplementary material for: Critical role of DEK and its regulation in tumorigenesis and metastasis of hepatocellular carcinoma
Source: Oncotarget. 2016 Apr 4;7(18):26844–55. doi: 10.18632/oncotarget.8565 (PMC5042019; doi:10.18632/oncotarget.8565)
Supplement: Supplementary file 1 [file oncotarget-07-26844-s001.pdf]

SUPPLEMENTARY FIGURES

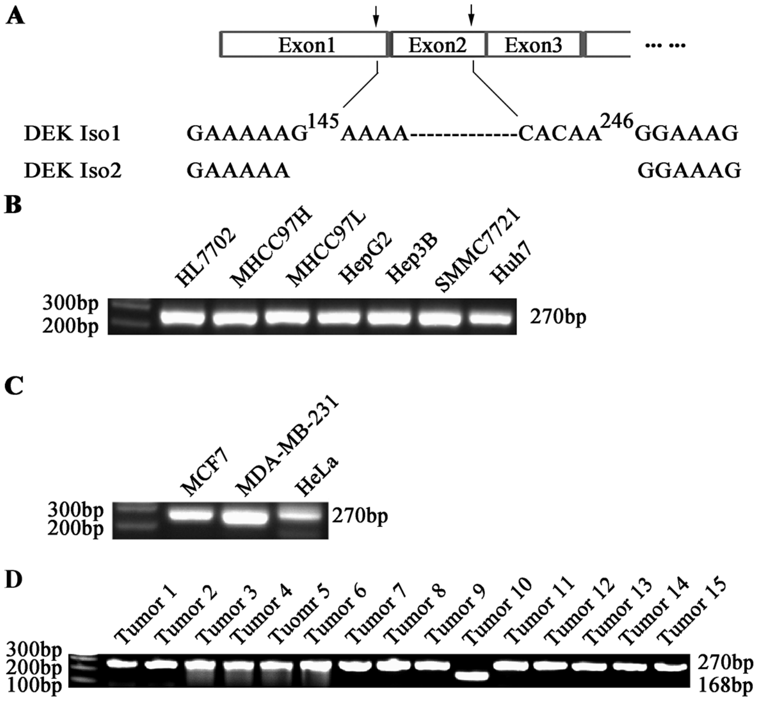

Supplementary Figure S1: Isoform 1 is the most frequently expressed DEK isoform in HCC cell lines and clinical specimens. A. Sequence comparison of isoform 1 (Iso1) and 2 (Iso2). **B–D.** RT-PCR was used to detect the presence of DKE isoforms in HCC cell lines (B) and other cell lines (C), and clinical HCC tissue samples (D).

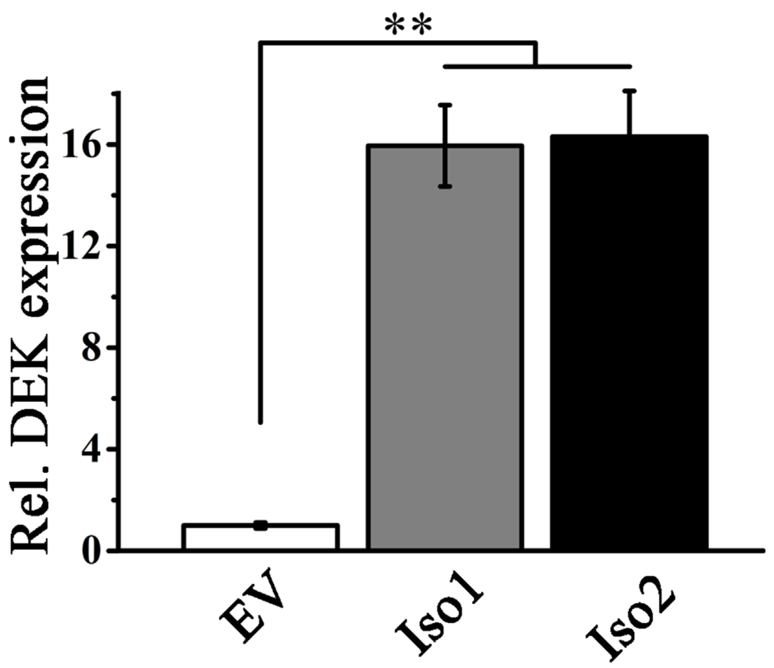

Supplementary Figure S2: RT-qPCR was performed to detect the overexpression effect of DEK in MHCC97L cells. Data are represented as mean ± SEM. **P<0.01.

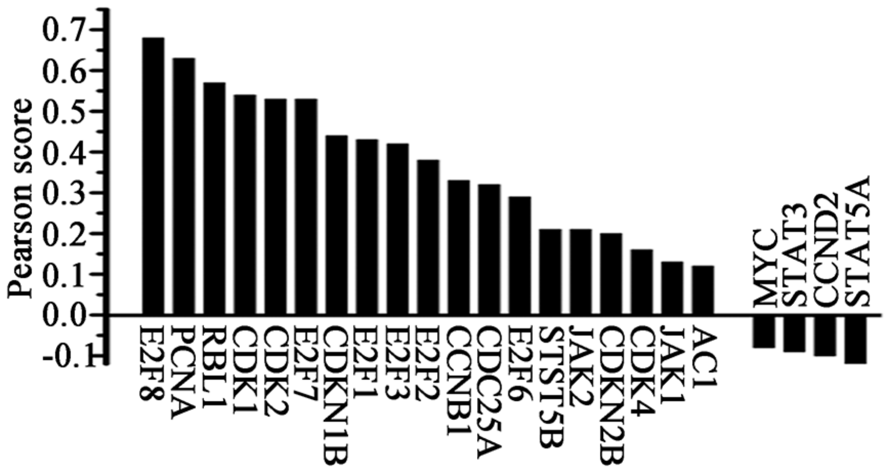

Supplementary Figure S3: Statistical analysis by cBioPortal was performed to determine the expression correlation between DEK and cell cycle related genes (Pearson's correlation coefficient >0.1 or <-0.1, *P<0.05).

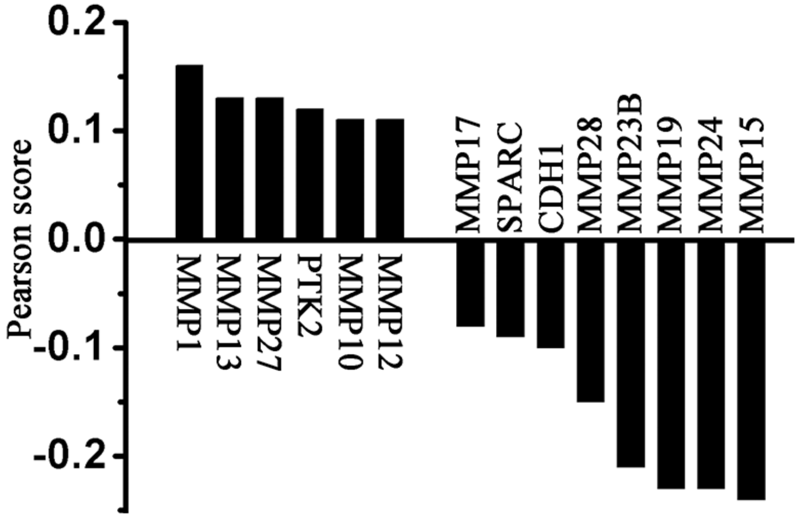

Supplementary Figure S4: Statistical analysis by cBioPortal was performed to determine the expression correlation between DEK and genes involved in migration (Pearson's correlation coefficient >0.1 or <-0.1, *P<0.05).

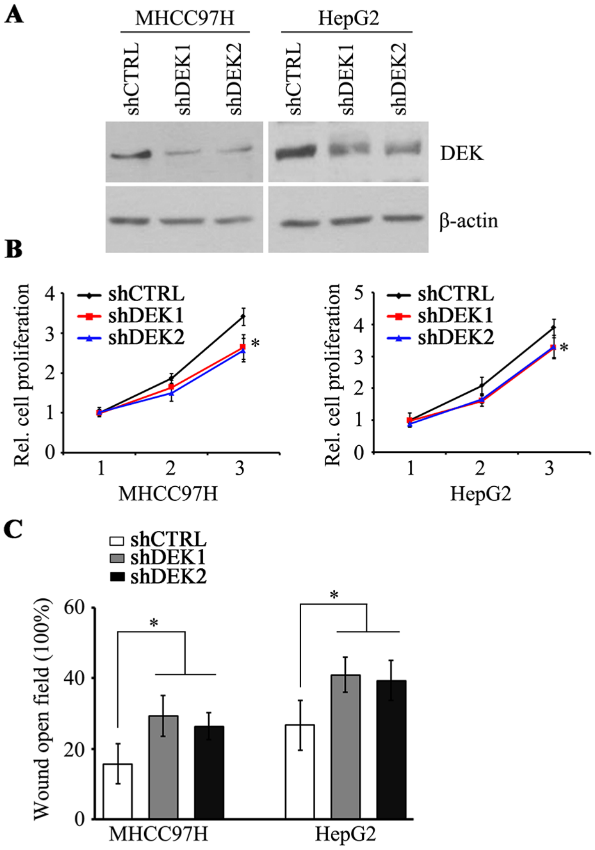

Supplementary Figure S5: DEK knockdown inhibits cell proliferation and migration in MHCC97H and HepG2 cells. **A.** Western blotting was performed to detect the knockdown effect by shRNA against DEK (shDEK-1 and shDEK-2). The non-target shRNA-expressing cells (shCTRL) were the knockdown control cells. **B.** MTS assay was performed to determine the cell proliferation when DEK was knocked down in MHCC97H and HepG2 cells. **C.** Wound-healing assay was employed to determine the migration of MHCC97H and HepG2 cells in response to DEK depletion. Cells were monitored within 24 hours to evaluate the rate of migration into the scratched area. Data are represented as mean ± SEM. *P<0.05.
